# Compressive behaviour of anisotropic mycelium-based composites

**DOI:** 10.1038/s41598-022-10930-5

**Published:** 2022-04-27

**Authors:** Adrien Rigobello, Phil Ayres

**Affiliations:** Centre for IT and Architecture, Royal Danish Academy, 1435 Copenhagen, Denmark

**Keywords:** Biomaterials, Fungal biology

## Abstract

Mycelium based composites (MBC) exhibit many properties that make them promising alternatives for less sustainable materials. However, there is no unified approach to their testing. We hypothesise that the two-phase particulate composite model and use of ASTM D1037 could provide a basis for systematisation. An experimental series of MBC were produced using four substrate particle sizes and subjected to compression testing. We report on their effect over Young’s modulus and ultimate strength. We extend the study by investigating three anisotropic substrate designs through orientated fibre placement as a strategy for modifying compressive behaviour. We find that the two-phase particulate model is appropriate for describing the mechanical behaviour of MBC and that mechanical behaviour can be modified through anisotropic designs using orientated fibres. We also confirm that fibre orientation and particle size are significant parameters in determining ultimate strength.

## Introduction

Mycelium-based composites (MBC) are being investigated in design and materials engineering by leveraging the saprotrophic lifestyle of ligninolytic fungi, taking inspiration in the XIXth to early XXth century method of fungal strain transfer by lignocellulosic solid-state cultivation^1^. Because MBC cultivation protocols can be based on virtually any substrate containing organic polymers such as lignin, hemicellulose and cellulose, and as they instrumentalise a range of widely available basidiomycota, this class of composite shows potential in obtaining viable products for a variety of uses. Furthermore, MBC conform to circular economy production principles, are expected to be biodegradable, and are assumed to have a low environmental impact in regards to Life-Cycle Assessment (LCA). Lignocellulosic substrates cover a variety of geometries and chemical profiles, from industrial grade dusts and particles to supplies of irregular shavings, from grain husks to straws; this variety of supplies has led to the emergence of a rich craft in MBC production. However, this poses a challenge in systematically understanding the behaviour of this new class of materials. We argue that rationalising and systematising approaches to analysing their complexity is necessary to actualise their potential and facilitate market readiness.

No analytical model has been previously proposed for MBC. We hypothesise that they qualify as two-phase particulate composites with the fungal mycelium acting as the matrix, and the substrate, with a high particle content ratio and randomly orientated, acting as the dispersed phase. Because the mechanical response of the fungal mycelium that binds particles together acts as a foam^2^, the composite stiffness is primarily driven by the substrate composition with angular particles. Previous studies of the failure mode of two-phase particulate composites have extensively investigated particle dewetting and their interfacial interactions for high particle content ratios^3^. Fourier-Transform Infrared (FTIR) spectrometry was used to qualify the materials used as principal substrate and fibre addition, the mycelium of *G. lucidum*, and *G. lucidum* colonised beech wood. This study then focuses on the influence of particle size on the compressive behaviour of MBC. The study is then extended to examine the influence of orientated fibres for modifying mechanical behaviour through anisotropic design. Three granulations of beech wood from 0.5 to 12.0 mm are used.

## Materials and methods

### Standard reference for specimen design

A variety of experimental designs are being used in the field of MBC research and engineering, as there is a current lack of unified approach to the material description. Few studies consider evaluation standards for MBC; among them, ASTM D3501 for wood-based structural panels in compression has been referenced^4^, a standard designed for plywood, wafer-board, orientated strand board, and composites of veneer and of wood-based layers, with use of 2:1 (D:h) cylindrical specimens in the study, instead of rectangular cross-section as the standard advises; ASTM D695 for rigid plastics was also referenced^5^, with a recommended 1:2 (D:h) ratio for cylindrical samples, but used with a diameter of 100mm and thickness of 23mm in the study; ASTM C67 destined to brick and structural clay tile was referenced in a comparative study against clay bricks^6^, but without following the standard recommendations; and ASTM D2166-13 for cohesive soil was referenced^7^, but deviating from the standard in the study. In requiring the largest particle to be smaller than one tenth of the specimen diameter, the latter ASTM exemplifies the instrumental role these standards can play in systematically investigating materials based on previous studies. The variety of specimen geometries found in the state of the art and lack of consistent recourse to the standards, challenges the portability of, and comparability between, experimental results.

Hypothesising that the best fit model for MBC is as two-phase particulate composite with a high particle content ratio, with particles randomly orientated, and, for this study, with sizes in the 0.5–12.0 mm range, we identify ASTM D1037 for assessment of wood-base fibre and particle panel materials mechanical properties as the most appropriate material standard for specimen and experimental plan design. We report compression parallel to surface evaluation, for which the short-column method has been chosen as the specimens have of a nominal thickness above 25 mm. They are parallelepipeds of 1:1:4 ratio, the nominal dimensions are 36 $\times$ 36 $\times$ 144 mm and the dimensions of the dried specimens are 34 $\times$ 34 $\times$ 140 mm. Our experimental plan investigates the effect of granulate sizes and reinforcement strategies over the compressive Young’s modulus and ultimate compressive strength.

### Principal substrates

The principal substrates of the specimens originated from European beech wood (*Fagus sylvatica*). To investigate the effect of particle sizes over the compressive behaviour, we used three granulations (small, medium, large): 0.5–1.0 mm (Räuchergold type HB 500/1000, J. Rettenmaier & Söhne GmbH + Co KG, Rosenberg, Germany), 0.75–3.0 mm (Räuchergold type HB 750/2000, J. Rettenmaier & Söhne GmbH + Co KG, Rosenberg, Germany), and 4.0–12.0 mm (Räuchergold type KL 2/16, J. Rettenmaier & Söhne GmbH + Co KG, Rosenberg, Germany). A fourth particle type was added to the experimental plan, as a 1:1:1 volume ratio mix of the three granulations.

### Fibre compositions

Longitudinal fibres were introduced in a specimen series by using common reed fibres (*Phragmites australis*; Tækkemand Chresten Finn Guld, Køge, Denmark). Eight to ten fibres of 1 mm ± 0.5 diameter were chosen so as to balance their dimensional variability and positioned in two layers separated by 10 mm of principal substrate. Fibres perpendicular to compressive stress were studied with use of 6 mm diameter by 32 mm length rattan fibres (*Calamus manan*; B.V. INAPO, Bloemendaal, Netherland). They were positioned regularly within the principal substrate as two layers of fibres, centred in the specimen thickness and separated by a 10 mm layer of principal substrate. It is common in MBC design practices to have mycelium grown externally on the outer boundaries of the specimens^4^. In the context of this study, no external mycelium was grown so as to observe the effect of granulate sizes and reinforcement strategies without introducing a specimen geometry bias. We identify this bias as critical for the reproducibility of experiments as the characteristics of the external mycelium mat is never found to be reported in the state of the art. In this study, a jacketing strategy has been integrated to study the effect of boundary reinforcement with a reproducible method. Across granulate sizes, a hemp-based hessian jacket (*Cannabis sativa* subsp. *sativa*; NEMO Hemp jam web 370 g/m$^{2}$, Naturellement Chanvre, Echandelys, France) was introduced on the specimen length. The study complies with relevant institutional, national, and international guidelines and legislation regarding the use of plant materials.

### Fungal species

*Trametes* spp., *Ganoderma* spp., and *Pleurotus* spp. are among the most frequently cited families in MBC design^8^; *Schizophyllum commune* is a less investigated species but finds a growing interest^9^, and *Irpex lacteus* has been used previously^7^. 565 carbohydrate-active enzyme families (CAZymes) have previously been assigned to the *Ganoderma lucidum* species^10,11^, representing the widest array from hydrolytic enzymes (hydrolysis of hemicellulose, pectin), to oxidoreductases (laccases, ligninolytic peroxidases and peroxide-generating oxidases), to cellobiose dehydrogenase. Because this species is considered a very versatile ligninolytic fungus, in that it can exploit various strategies for the breakdown of lignin and can ultimately degrade all components of lignocellulosic compounds, it was selected for implementing the experimental plan. A millet-grown spawn of ligninolytic species *G. lucidum* (reference M9726) was acquired from Mycelia BVBA (Nevele, Belgium). The spawn was stored at a constant 4 $^\circ$C and 65% relative humidity (RH).

### Specimen preparation

The moisture content (MC) within cell walls as bound water, and outside cell walls in wood void structure as capillary water or vapour, is critical for understanding and predicting fungal activity^12^. In MBC production, lignocellulosic substrates composed of particle or fibres have a MC that is homogeneously prepared at 55–70%^4,13^, and the use of mineralized to sterile demineralized water has been documented as a moisturising mean^4,13,14^.

For this study, the principal substrates, fibres and hessian were prepared at 40% MC with mineralized water, and sterilised at 121 $^\circ$C for 15 min. The principal substrates were then mixed with 16 wt% *G. lucidum* spawn and incubated in PP filtered bags (PPD50/REH4+1/V22-49, SacO2, Deinze, Belgium) for 7 days at 25 $^\circ$C in the dark. Once colonised, the principal substrates were massaged to break them down and formed with the fibres and hessian into aerated PETG moulds. The formed specimens were incubated for 21 days at 25 $^\circ$C in the dark, then oven dried for 48 h at 60 $^\circ$C. The dried specimens were stored at 4 $^\circ$C and 65% RH prior to testing.

### Compressive behaviour characterisation

The use of seismic waves to characterise the mechanical behaviour of MBC has been reported in the literature as an alternative to conventional uniaxial load testing^7^. This method has become common in geological and civil engineering, and offers the benefit of being non-destructive. However, the anisotropic nature of the composite matrix (the mycelium), together with is its high elasticity, causes waves to attenuate irregularly. Furthermore, MBC have such a high porosity and a high variation in particle sizes and distribution that gaining accurate measurements would be challenging. This is evidenced in the literature by a larger standard deviation in results using this process applied to homogeneous MBC^7^. In the context of this study, load testing was performed on a Mecmesin MultiTest-dV testing bench equipped with a 2500 N load sensor, with a loading speed of 1.0 mm/min. Young’s modulus and ultimate compressive strength were calculated following ASTM D1037.

### Chemical analysis

Fourier-Transform Infrared (FTIR) spectrometry has been used previously for analysing the lignocellulosic profiles of substrates and their relation to fungal degradation patterns^4,15–17^, with the benefit of requiring a limited specimen preparation, and spectra shape and frequencies being directly related to microscopical physical quantities and hence prepared for interpretation^18^. FTIR spectrometry was conducted in this study on a single reflection diamond Attenuated Total Reflectance (ATR) Agilent 4500a FTIR (Santa Clara, USA). The acquisition resolution was 4 cm$^{-1}$ with 16 scans per specimen, for a band between 4000 and 650 cm$^{-1}$. We corrected the baseline of FTIR spectra following the adaptive iteratively reweighted Penalised Least Squares (airPLS) method^19^, and spectra normalization was done with amide I/II band envelopes^20^. Four samples were isolated from *G. lucidum* colonised beech wood specimens, their spectra were averaged for analysis. The other specimens were tested with one replicate.

## Chemical analysis

**Figure 1:**
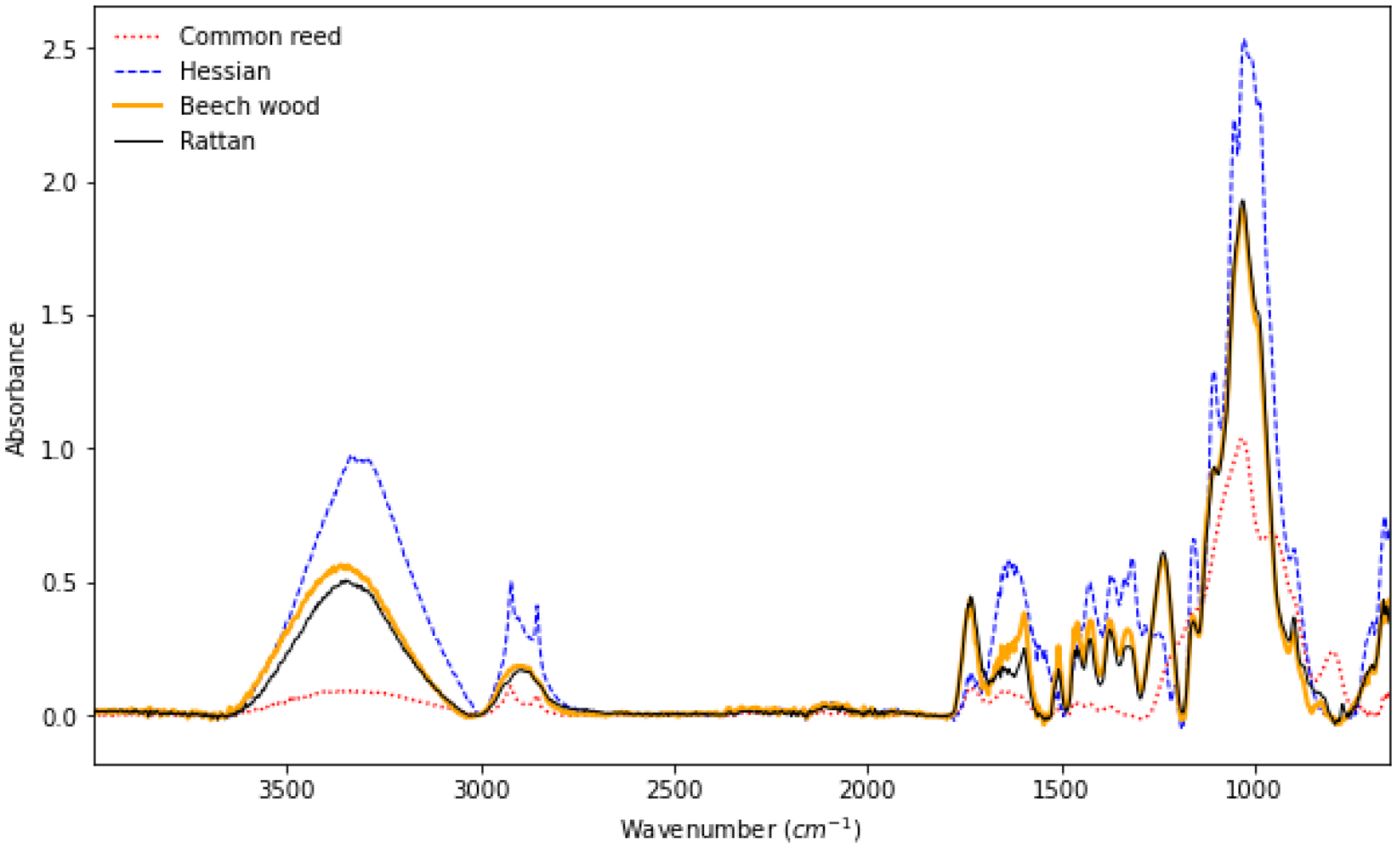
FTIR spectra of hemp-based hessian, beech wood, rattan, and common reed fibres.

To serve as controls, we used FTIR spectrometry to characterise the four materials used in the composite design (Fig. 1). The materials were hemp-based hessian, beech wood, rattan, and common reed. Beech wood and rattan spectra display a chemical profile that is very similar, with the exception of peaks at 1123 cm$^{-1}$ and 1160 cm$^{-1}$, and the 1300–1500 cm$^{-1}$ region. This indicates a slightly higher content of cellulose, hemicellulose and lignin in our tested beech wood specimen (C–O stretching, C–O–C asymmetrical stretching, C–H deformation, COOH groups symmetrical stretching, symmetric C–H bending, CH$_2$ deformation stretching, CH$_3$ asymmetrical angular vibration, vibrational mode of amide C–O stretching)^21–23^. Common reed displays a minimal amount of lignin and hemicellulose compared to our other samples, while the peak at 890 cm$^{-1}$ is associated with C–O–C stretching at the $\beta$–(1$\rightarrow$4)–glycosidic linkages of amorphous cellulose^24^. The hessian displays distinctive peaks at 707 cm$^{-1}$, 890 cm$^{-1}$, 1060 cm$^{-1}$, 1316 cm$^{-1}$, and 1430 cm$^{-1}$ in the fingerprint region, and 1640 cm$^{-1}$, and 2921 cm$^{-1}$. The 750–680 cm$^{-1}$ and 1680 – 1630 cm$^{-1}$ regions (C=O streching) are associated with primary and secondary amides in hemp (amide V: C–N and N–H vibrations)^25^. Primary amides in hemp are amino acids, fatty acids, and steroids, which contribute to the 3500–3000 cm$^{-1}$ region. The 1310–1230 cm$^{-1}$ region (C–N stretching) is associated to secondary amides, such as cannabinoids, flavonoids, stilbenoids, terpenoids, alkaloids, and lignans^26^. The peak at 2921 cm$^{-1}$ is associated with alkyl C–H groups^27^.

**Figure 2:**
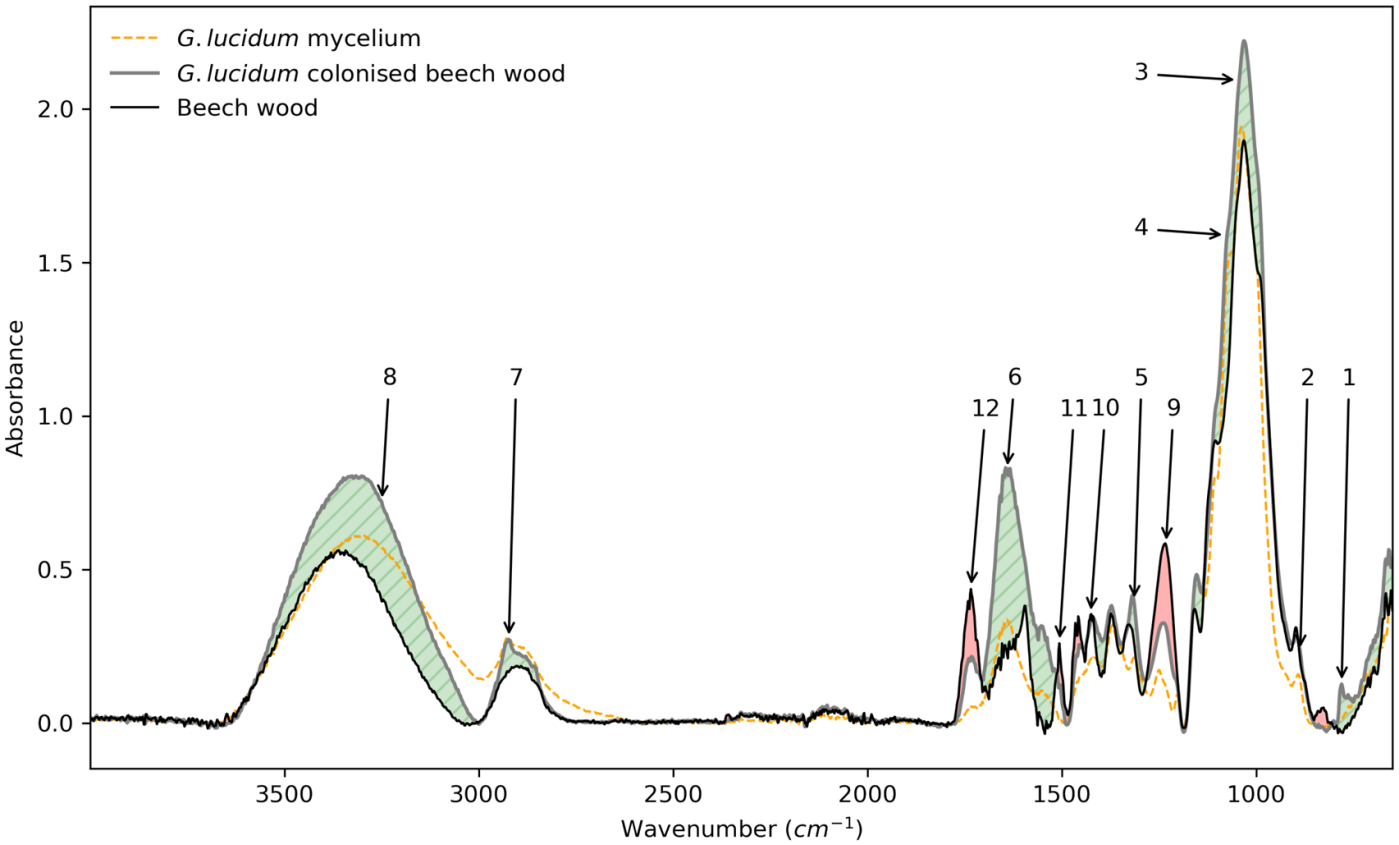
FTIR spectra of *G. lucidum* colonised beech wood, *G. lucidum* mycelium, and beech wood. Green areas represent increased values in mycelium-colonised specimens (peaks 1 to 8), red areas are decreased values in mycelium-colonised specimens (peaks 9 to 12).

Four samples were isolated from *G. lucidum* colonised beech wood specimens after they were used for load testing. Their spectra were averaged and are presented on Fig. 2 along with the beech wood spectrum, and a sample of *G. lucidum* mycelium. Peaks at 886 cm$^{-1}$, 1075 cm$^{-1}$ and 1160 cm$^{-1}$ are characteristic of (1$\rightarrow$3)– and (1$\rightarrow$6)–$\beta$–glucans that are present in the fungal cell wall (identified as [2], and [4] on Fig. 2). The peaks [1] and [3] at 780 cm$^{-1}$ and 1043 cm$^{-1}$ are also associated with $\beta$–glucans^28^. Chitin is identified at peak [5] 1313 cm$^{-1}$(amide III: C–N stretching), which also affects the 1640 cm$^{-1}$ region [6] alongside the presence of peptides and secondary metabolites (aromatic rings and conjugated alkenes). The peak [7] at 2922 cm$^{-1}$ is representative of chitin and ergosterol (C–H stretching)^29^. The 3600–3000 cm$^{-1}$ region (peak [8]) is considered to be influenced by residual water and entrapped CO$_2$ (O–H and N–H stretching). Finally, peaks [9] to [12] represent decreases at 1231 cm$^{-1}$, 1425 cm$^{-1}$, 1506 cm$^{-1}$, and 1733 cm$^{-1}$. They are associated with lignin and xylan breakdown (syringyl ring breathing and C–O stretching, C=C stretching vibration in aromatic ring), and cellulose (peak [11]) and hemicellulose (peak [11] and [12]) breakdown is observed (CH$_2$ scissor vibration, C=O stretching)^30^. To evaluate the lignocellulosic changes undertaken during *G. lucidum* activity quantitatively, the band ratio indices at 1231 cm$^{-1}$, 1425 cm$^{-1}$, and 1506 cm$^{-1}$ were calculated from the 2921 cm$^{-1}$ band^31^ for beech wood and *G. lucidum* colonised beech wood as:

1 $$\begin{aligned} \frac{I_{n}}{I_{2921}}, \end{aligned}$$

Where $I_{n}$ is the specific band intensity and $I_{2921}$ the band intensity at 2921 cm$^{-1}$. The band ratio at 1231 cm$^{-1}$ went from 1.58 in beech wood to 0.61 in *G. lucidum* colonised beech wood; the band ratio at 1425 cm$^{-1}$ went from 0.96 in beech wood to 0.63 in *G. lucidum* colonised beech wood; the band ratio at 1506 cm$^{-1}$ went from 0.71 in beech wood to 0.24 in *G. lucidum* colonised beech wood; the band ratio at 1733 cm$^{-1}$ went from 1.19 in beech wood to 0.41 in *G. lucidum* colonised beech wood. We can therefore observe that *G. lucidum* had a preference in breaking down lignin and xylan at 1231 cm$^{-1}$ compared to cellulose and hemicellulose at 1425 cm$^{-1}$ (2.94:1), which is confirmed by the ratios at 1506 cm$^{-1}$ for lignin (1.42:1), and 1733 cm$^{-1}$ for hemicellulose (2.36:1). The CH$_2$ scissor vibration corresponding to the peak at 1425 cm$^{-1}$ reflecting both cellulose and hemicellulose, the present decrease might be primarily related to hemicellulose breakdown. This preference of *G. lucidum* for lignin and hemicellulose is consistent with findings reported in the literature^10^.

## Compressive behaviour

**Figure 3:**
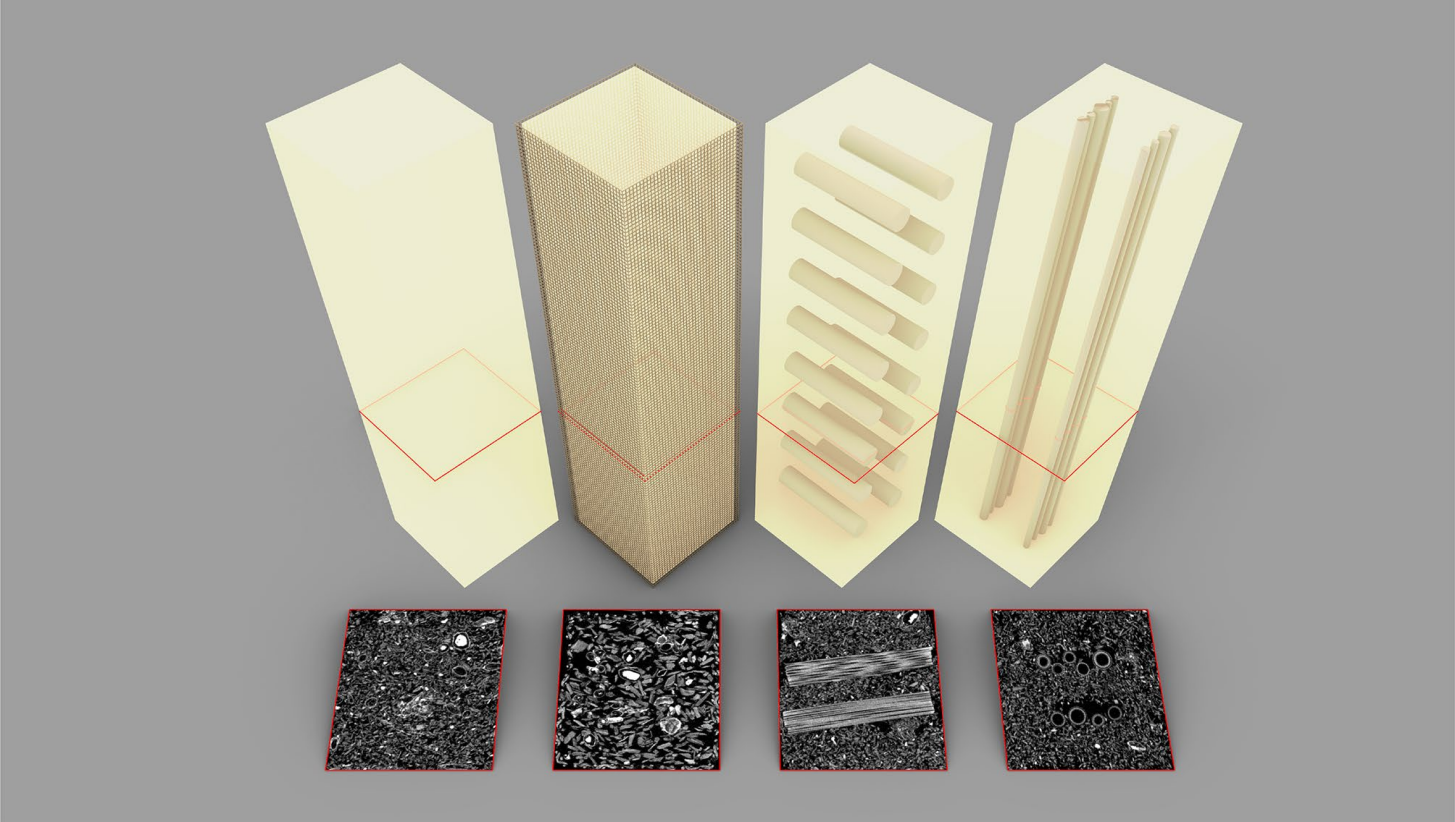
Fibre placement strategies and their sectional CT scan (left to right): control (BS), jacketing coaxial to load (BM_H), fibres perpendicular to load (BS_R), fibres coaxial to load (BS_V).

**Figure 4:**
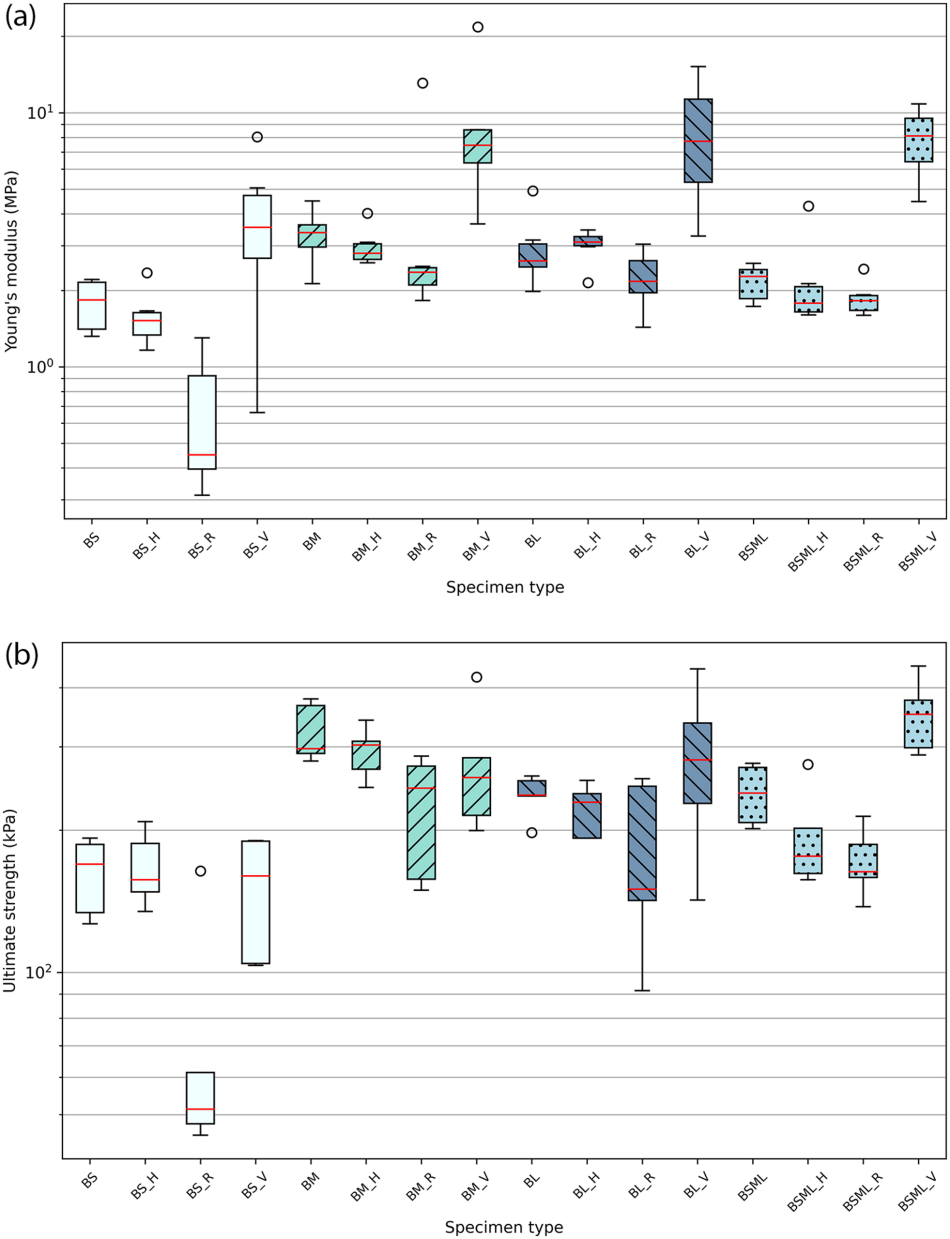
Box plots for Young’s modulus results (**a**) and ultimate strength results (**b**).

**Figure 5:**
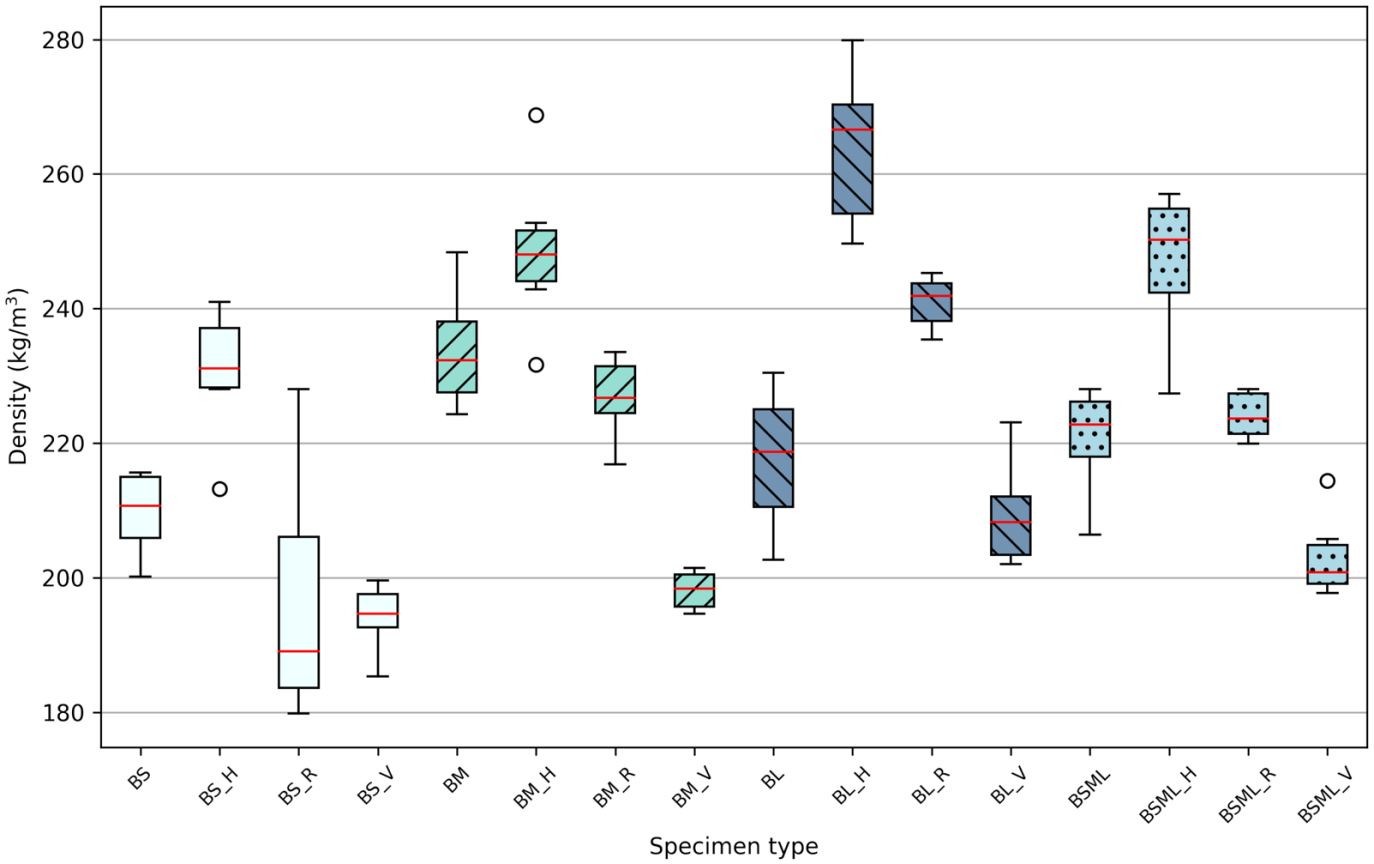
Box plots for dried specimen densities.

We investigated the effect of particle sizes on the mechanical behaviour in compression of MBC using four levels of granulation: small (BS family), medium (BM family), large particles (BL family), and a 1:1:1 volume ratio mix of the three previous granulations (BSML family). A second parameter was introduced to investigate the anisotrope modification of MBC. Three typologies of fibre composition were implemented in the experimental plan: hessian jacketing coaxial to the load case (H), unidirectional rattan fibres perpendicular to the load case (R), and unidirectional common reed fibres coaxial to the load case (V). Isotropic controls were added for each level of granulation (BS, BM, BL, BSML specimen types in the figures). Fig.3 illustrates the three typologies alongside the control. Experimental parameters per specimen type and resulting mean density, mean Young’s modulus and mean ultimate strength are presented in Table 1. Box plots of the results for Young’s modulus and ultimate strength are presented in Fig. 4, and box plots for densities are reported in Fig. 5.

**Table 1:** Summary of specimen types parameters, resulting dried densities, and compressive properties.

Specimen type	Granulate size (mm)	Fibre composition	Mean density (s.d.)	Mean Young’s modulus (s.d.)	Mean ultimate strength (s.d.)
BS	0.5–1.0	Control	209.67 kg/m$^{3}$ (6.47)	1.79 MPa (0.41)	171.86 kPa (36.54)
BS_H	0.5–1.0	Hessian jacketing	230.48 kg/m$^{3}$ (9.88)	1.58 MPa (0.42)	175.79 kPa (34.38)
BS_R	0.5–1.0	Rattan perpendicular to load	196.59 kg/m$^{3}$ (19.01)	0.66 MPa (0.42)	89.06 kPa (58.49)
BS_V	0.5–1.0	Common reed coaxial to load	194.12 kg/m$^{3}$ (5.09)	3.88 MPa (2.51)	146.85 kPa (39.26)
BM	0.75–3.0	Control	233.87 kg/m$^{3}$ (9.04)	3.32 MPa (0.80)	306.38 kPa (57.64)
BM_H	0.75–3.0	Hessian jacketing	248.70 kg/m$^{3}$ (12.20)	2.99 MPa (0.54)	298.85 kPa (35.47)
BM_R	0.75–3.0	Rattan perpendicular to load	226.77 kg/m$^{3}$ (6.19)	4.02 MPa (4.45)	232.30 kPa (62.85)
BM_V	0.75–3.0	Common reed coaxial to load	198.14 kg/m$^{3}$ (2.89)	9.21 MPa (6.42)	270.93 kPa (79.76)
BL	4.0–12.0	Control	217.60 kg/m$^{3}$ (10.58)	2.96 MPa (1.04)	245.60 kPa (30.31)
BL_H	4.0–12.0	Hessian jacketing	264.05 kg/m$^{3}$ (11.97)	3.01 MPa (0.46)	223.93 kPa (25.88)
BL_R	4.0–12.0	Rattan perpendicular to load	240.98 kg/m$^{3}$ (3.91)	2.24 MPa (0.58)	180.88 kPa (64.75)
BL_V	4.0–12.0	Common reed coaxial to load	209.47 kg/m$^{3}$ (7.90)	8.50 MPa (4.56)	290.86 kPa (100.83)
BSML	0.5–12.0	Control	220.59 kg/m$^{3}$ (8.12)	2.17 MPa (0.36)	237.09 kPa (31.73)
BSML_H	0.5–12.0	Hessian jacketing	246.85 kg/m$^{3}$ (11.29)	2.20 MPa (1.04)	194.48 kPa (48.47)
BSML_R	0.5–12.0	Rattan perpendicular to load	224.09 kg/m$^{3}$ (3.55)	1.87 MPa (0.30)	171.44 kPa (26.13)
BSML_V	0.5–12.0	Common reed coaxial to load	203.08 kg/m$^{3}$ (6.24)	7.89 MPa (2.41)	338.75 kPa (65.39)

### Jacketing coaxial to load

The introduction of the hessian jacket offers a contrasting illustration of the effect of the mycelial mat usually grown on the external boundary of MBC. We observe that the dispersion of Young’s modulus results across all specimen families is reduced compared to their controls with the exception of the BL family. The jacketing also affects the dispersion of results in ultimate strength in the case of the BS, BM, and BSML families, with a reduction of the deviation between the first and third quartiles. The containment of stress applied to the specimens within tight boundaries forces the arrangement of the particles within, restricting the ability for particles to arrange freely. Jacketed specimens have an average reduction of 0.12 MPa to the controls as per Young’s modulus (s.d. 0.19), and an average decrease of 16.97 kPa to the controls as per ultimate strength (s.d. 20.05). The jacket has two important advantages: it offers a durable alternative to low-ductility mycelial mats usually grown on the external boundary of MBC, and we hypothesise that it can substantially contribute to an increase in fracture resistance performance in shearing and bending load cases.

### Fibres perpendicular to load

**Figure 6:**
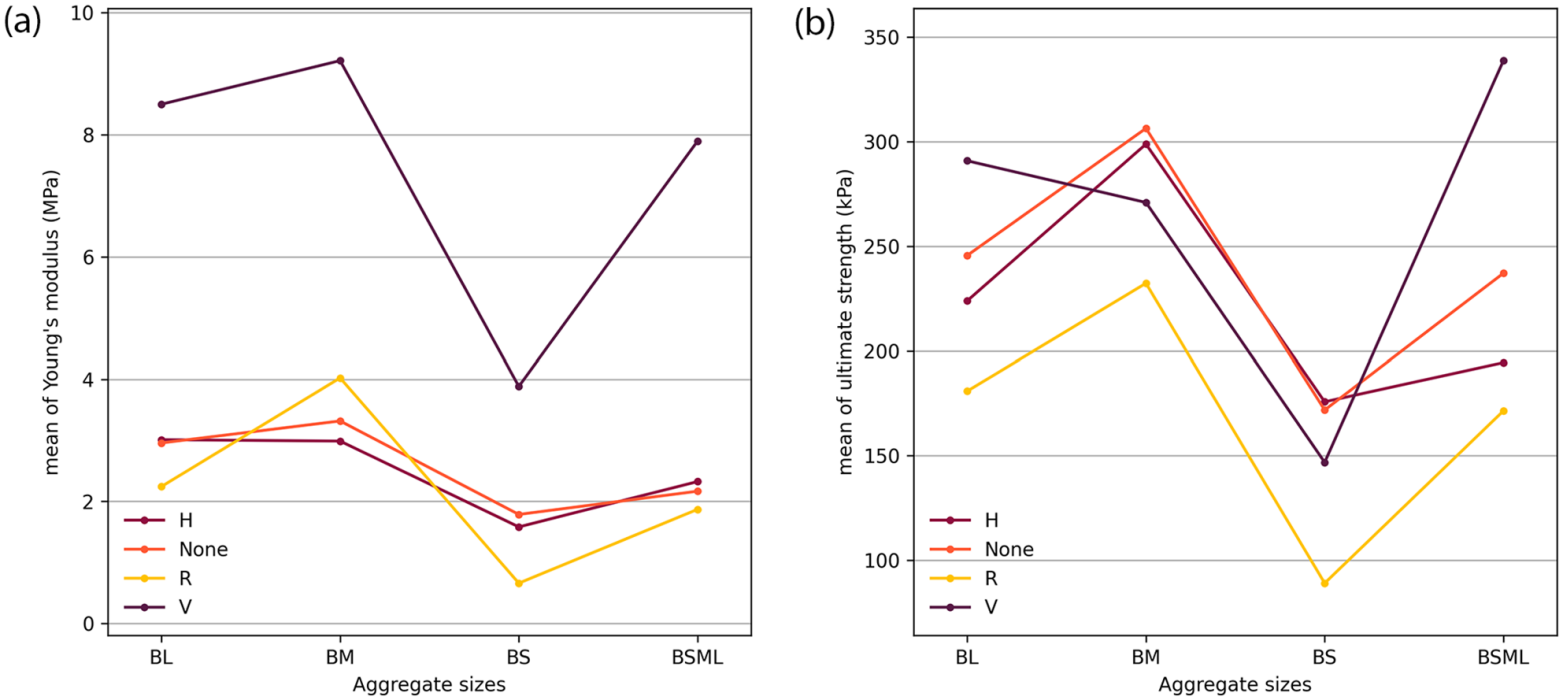
Parameters interaction graph for Young’s modulus (**a**) and ultimate strength (**b**).

Specimens supplemented with rattan fibres display a lower performance across particle sizes considering their median in Young’s modulus and ultimate strength. The mean ultimate strength follows the performance of the mean of the controls (Fig. 6) with an average reduction of 71.81 kPa (s.d. 8.45). This suggests that, should the production conditions of such MBC improve to reduce the dispersion of results and increase the material behaviour predictability, introducing strategically parsed weakness points in composites could find a use with calibrated materials by tuning their failure mode.

### Fibres coaxial to load

Common reed fibre reinforced specimens resulted in the largest standard deviations in Young’s moduli (reported in Table 1), especially in the BM and BL families. This is due to the fibres having partially misaligned to the load case axis during specimen production. Nevertheless, results suggest that MBC can be successfully stiffened with regards to their use case. The effect of this stiffening on the ultimate strength is less obvious as we note that the smaller particles (BS and BM families) tend to perform better without reinforcement coaxial to load. This is a result of the inherent large displacement of the fibres within the specimens under stress due to their stiffness, thus initiating an early critical failure. The mean Young’s moduli (Table 1) display a clear improvement compared to the controls: we observe an average increase of a factor 2.86 (s.d. 0.6) between the mean of the controls and the mean of the fibre coaxial to load specimens. As per mean ultimate strengths, they improved in the BL and BSML families when compared to controls (respectively by a factor 1.18 and 1.43), but decreased in the smaller particles families BS and BM (respectively by a factor 0.86 and 0.88).

### Principal substrate particles

The use of smaller particles in MBC increases the surface area to volume ratio of what serves as a nutrient for the fungus, hence facilitating its access to it. Fungi also need air access to develop a mycelium, and space between particles, if one desires to have it synthesise a biomass that has a considerable effect over its mechanical properties. The small granulation essentially qualifies as a dust with particles size in the 0.5–1.0 mm interval, leaving minimal amounts of air between particles within the constrained boundaries of the specimen mould. The best performing BM family (as per ultimate strength) is composed of 0.75–3.0 mm particles, thus embedding particles of a comparable size to the BS dust, while containing particles that are up to six times as long. This understanding is nonetheless challenged by looking at the BS, BM, BL and BSML group densities (Table 1 and Fig. 5), where the BS group has the lowest resulting density.

Studying a material model composed of cylindrical particles with a length in 2–10 mm and diameter of 0.5–2 mm, a study concludes that the matrix phase of MBC is ruling the composite modulus^2^. This study avoids considering more particle shape parameters that have been shown to have a significant effect over the system behaviour; flakiness/flatness (thickness to width ratio), elongation (length to width ratio), sphericity (deviation from a sphere geometry), and roundness/angularity (angular sharpness) have been previously investigated in particle studies of granular materials^32^. It was reported that a 3:1 ratio of flaky particles content would be an approximate optimal for shear strength (depending on the system of study). This is related to cohesion being increased under stress due to particle interlocking. A higher particle angularity was reported to induce a decrease in elastic modulus, and an increase in ultimate strength. Shear strength was reported to increase with particle angularity. Increasing particle flakiness and angularity increases cohesion and abrasion. This leads to damage accumulation under repeated loads, resulting in strain accumulation^32^. This suggests that modifying composite behaviour does not only depend on mycelial expression, but should investigate substrate contribution too systematically.

**Figure 7:**
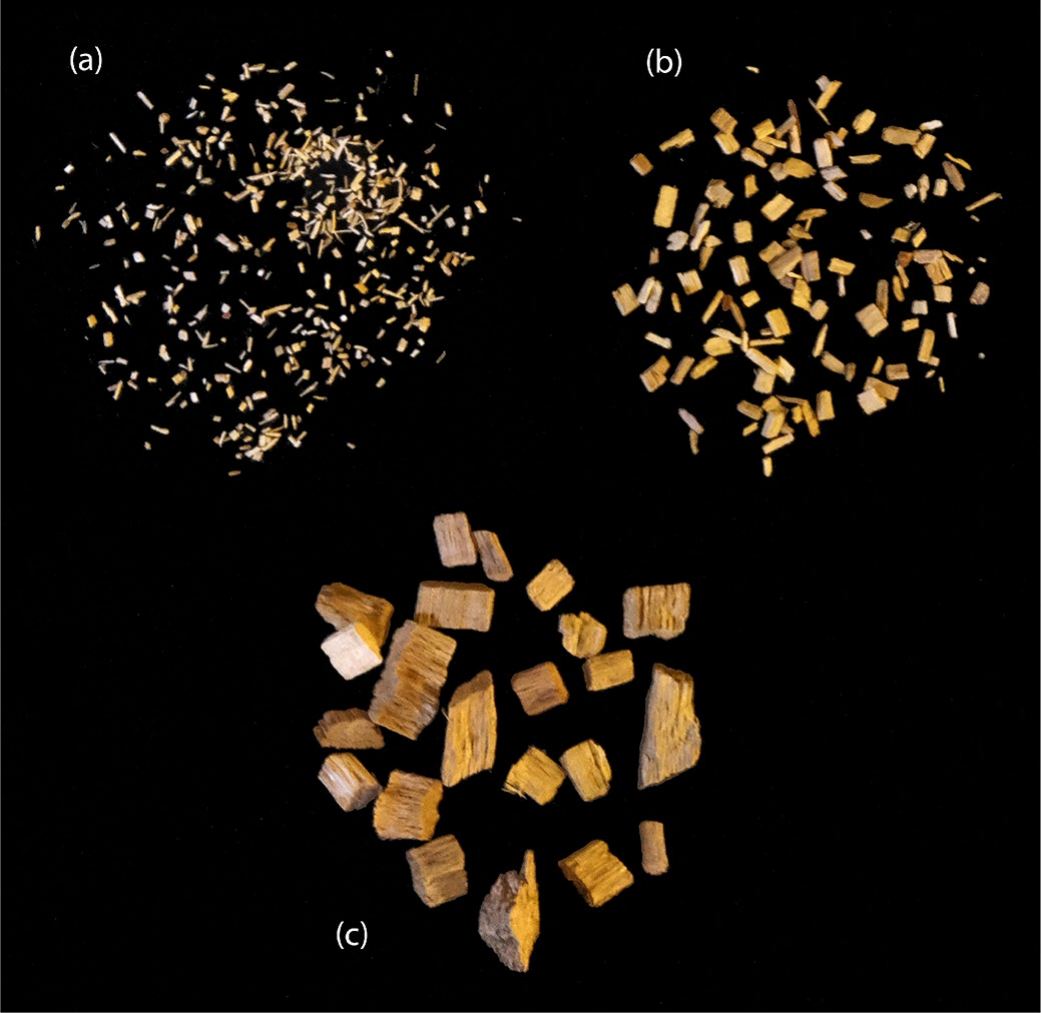
Beech wood particles of small (**a**), medium (**b**) and large (**c**) granulation used in this study.

While the BM group mean density is 11.54% higher and the most different to the BS group mean density, the BM mean elastic modulus is 85.48% higher than that of the BS group and 78.27% higher in ultimate strength. In this experiment, there is a correlation between an increased density and increased stiffness and strength. As the principal substrate used in these four groups is of the same nature and source, we can note that the particle volume fractions are directly correlated to the densities. It is worth noting too that the different levels of granulation result in different aggregate mechanical properties; on Fig. 7 we can notice the effect of comminution, smaller granulation (a) result in a higher content of short fibres with a lower bending stiffness, medium sized particles (b) display a content of not only fibre-type particles but also less elongated, more angular and bulky ones, contributing to increase their bending stiffness and interlocking potential under stress, and finally the larger granulates (c) display an increased flakiness and angularity to the medium ones. While the latter would be expected to result in higher stiffness and strength to the other groups because of their aggregate geometrical characteristics, the manufacturing of the specimens did not focus on particulate arrangements for this series and therefore these were randomly orientated and thus not optimised for interlocking.

Pure mycelium material response under tensile and compressive stress has been investigated and modelled^2^, and has been classified as an open-cell foam-like material. Pure mycelium of an undisclosed species in this study was reported to exhibit a Young’s modulus of 0.6–2 MPa in tension and compression, and an ultimate tensile strength of 0.1–0.3 MPa. So as to situate this report, a *P. ostreatus* mycelium has been reported to exhibit a Young’s modulus of up to 28 MPa for an ultimate strength of 0.7 MPa, and a *G. lucidum* mycelium a Young’s modulus of up to 12 MPa for an ultimate strength of 1.1 MPa^17^. Beech wood has a Young’s modulus of 11.9 GPa at 12% moisture content, and 9.5 GPa when green^33^. Beech wood particles are therefore important load-carrying members of the system and reduce the magnitude of stress experienced by the mycelial matrix. The plastic strain of the composite is contributed to only by particles in such composite, which is clearly exhibited in the range of results in Fig. 4. As introduced with the common reed and rattan containing specimens, the dewetting behaviour of the larger particles or reinforcements present in the composite is a principal contributor to damage nucleation. Furthermore, the shape, nature, and distribution of particles in a two-phase composite has been shown to have a substantial influence over the load transfer between members and hence their overall stiffness^3^. Moreover, while lignin is a primary contributor of strength parallel to grain, hemicellulose supports compression strength perpendicular to grain. Its decay greatly affects the structural integrity of wood and its hardness^34^.

**Figure 8:**
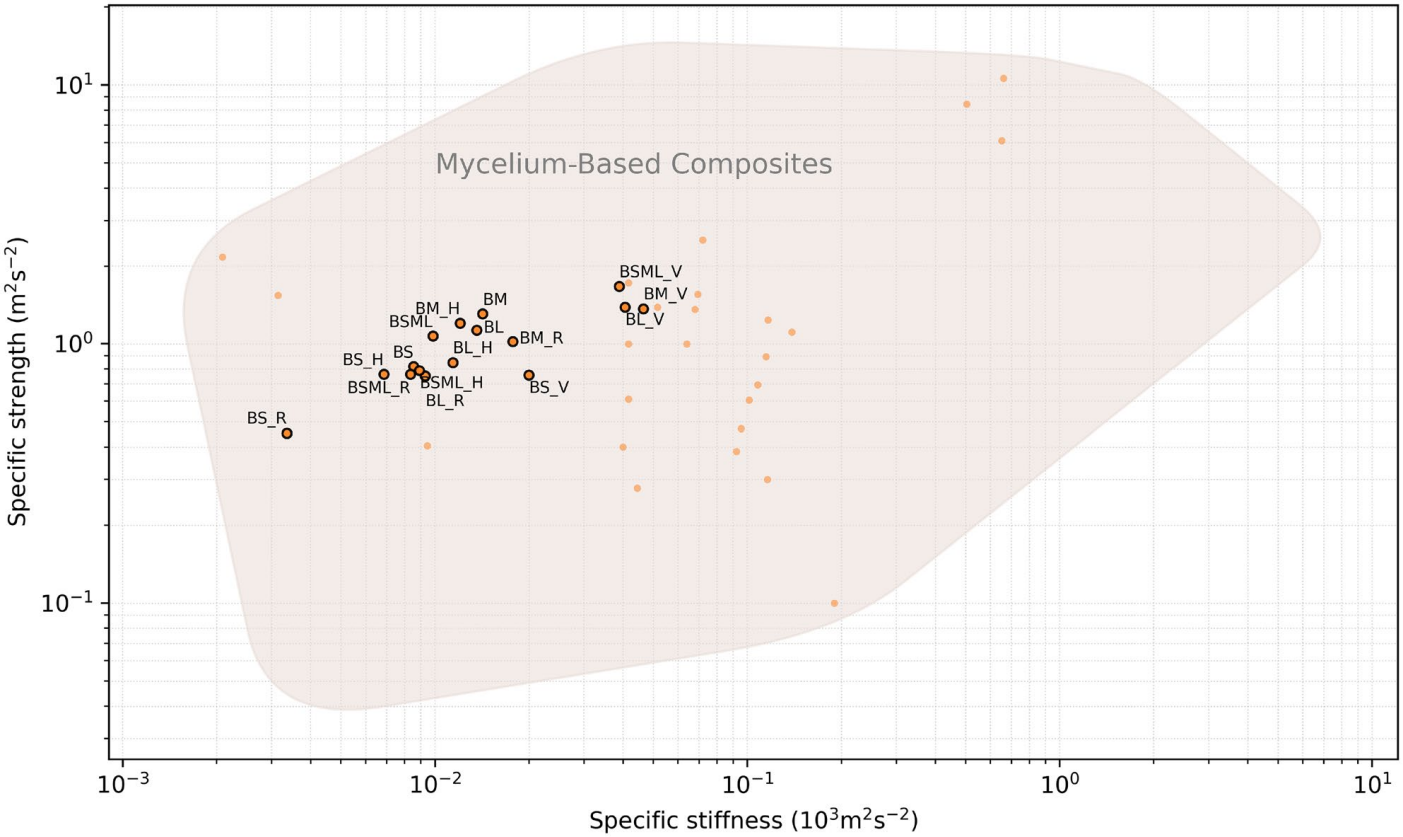
Specific strength results as a function of specific stiffness. Labelled data points: results from this study; unlabelled data points: reports from the state-of-the-art.

**Figure 9:**
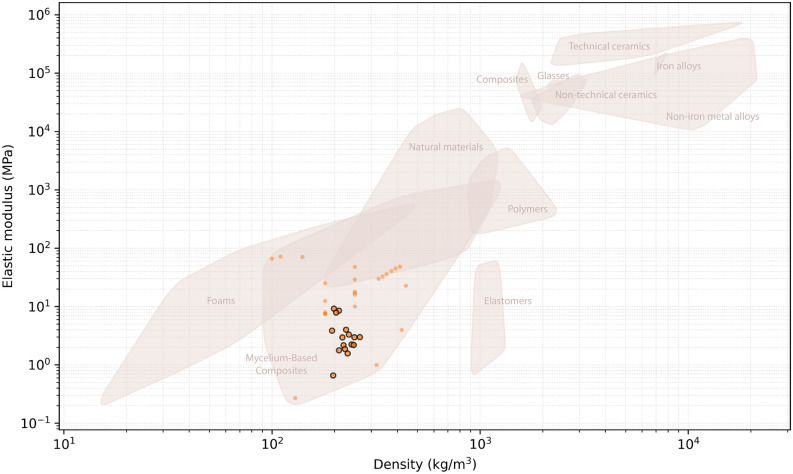
Young’s modulus results as a function of density. Circled data points: results from this study; uncircled data points: reports from the state-of-the-art.

The results of the experimental series are plotted as normalised by density in Fig. 8, and on an Ashby map for elastic and density (Fig. 9). In both figures compressive characterisation from the published MBC state-of-the-art are plotted^4–7,35–43^. These figures gather evidences produced with approximately ten fungal species, two studies having not disclosed the ones they used^6,36^. There are 69 data points gathered from thirteen journal and conference articles. These include articles reporting on strength and/or stiffness in compression; 6 data points had no density reported^7^. Only the reports with sufficient data are rendered on the figures.

While wood particles are of common use in MBC, other substrates have been used such as non woven cotton fibres^13^. A small number of studies have investigated the addition of non-organic aggregates to a lignocellulosic substrate for improving its stiffness, such as with carbonate sand^38^, and sand and gravel^44^.

## Statistical analysis

Six replicates were produced and tested for each of the specimen types, the distributions are two-tailed. The mean of Fisher’s defined kurtosis for Young’s modulus series is − 0.3328 (s.d. 0.9353) and − 1.0564 for ultimate strength (s.d. 0.5343). Fisher–Pearson’s skewness coefficient mean for Young’s modulus is 0.5834 (s.d. 0.8540), and 0.1733 for ultimate strength (s.d. 0.5137). The distributions are considered normal^45^, which was verified for ultimate strength and Young’s modulus results with the Shapiro–Wilk test (respectively *p* = 0.9224 and *p* = 0.0030, $\alpha$=0.001). Equality of variances was therefore controlled with the Levene test; Young’s modulus result variances are not equal (*p* = 1.3940e−05, $\alpha$=0.05), neither are ultimate strength ones (*p* = 0.0459, $\alpha$=0.05). Welch’s ANOVA was conducted for the two parameters: fibre placement for Young’s modulus and ultimate strength (respectively *p* = 0.0001 and *p* = 0.0013), and particle size for Young’s modulus and ultimate strength (respectively *p* = 0.0030 and *p* = 4.6462e−09). The mean values of specimen groups are significantly different ($\alpha$=0.005). Using the pairwise Games-Howell test we identified the most significant reinforcement to be the fibre coaxial to load against fibre perpendicular to load, the control, and hessian jacketing (all *p* = 0.001 as per Young’s modulus; respectively *p* = 0.030, *p* = 0.004, and *p* = 0.004 as per ultimate strength; $\alpha$=0.05). Continuing this test, we identified the most significant aggregate size to be the 0.5–1.0 mm interval (BS family) against the BM and BL families (respectively *p* = 0.029 and *p* = 0.036 as per Young’s modulus; all *p* = 0.001 as per ultimate strength; $\alpha$=0.05). The BS family had a significant difference to the BSML family as per aggregate size over ultimate strength (*p* = 0.001, $\alpha$=0.05), but not over Young’s modulus (*p* = 0.106, $\alpha$=0.05).

## Conclusions

Across the literature, we find that the lack of a unified approach in the use of analytical models and/or methodological approaches has resulted in inconsistency with specimen design, cultivation and testing protocols. This raises the question of portability and comparability of results. The adoption of the two-phase particulate composite model helped us identify ASTM D1037 as the most appropriate candidate to support the design of specimens and of the experimental plan. As a general observation, we found that specimens using particles in the 0.75–3.0 mm range resulted in a higher strength and stiffness in compression.

We extended this study to fibre placement strategies with three typologies: rattan fibres perpendicular to load, common reed fibres coaxial to load, and hessian jacketing coaxial to load. The addition of fibre coaxial to load and hessian jacketing had a significant effect over Young’s elastic modulus and ultimate strength ($\alpha$=0.05). Fourier-Transform Infrared (FTIR) spectrometry was used to qualify (1) the materials used as principal substrate and fibre addition, (2) the mycelium of *G. lucidum*, (3) and *G. lucidum* colonised beech wood. We found that the *G. lucidum* species degraded primarily lignin and hemicellulose before cellulose, in accordance with previous observations^10,46^.

Because of the wide range in particle sizes used and fibre composition typologies, the significant difference between specimen groups supports our hypothesis that the two-phase particulate model is suited for future MBC studies ($\alpha$=0.005). These studies might involve exploring a wider variety of particle shapes, natures, and distributions as these parameters have been shown to have a significant influence over the elastic and plastic behaviour of composites^3^. We demonstrated that the modifying of specimens could be attained with contrasting examples of coaxial reinforcement and perpendicular fracture initiators, with significant effect ($\alpha$=0.005). However, it should be noted that fibre placements were subjected to variability as fibres could partially misalign with the load axis or its perpendicular during production. This suggests that the standard deviation of the results can be reduced by improving the accuracy in manufacturing.

Specific strength and stiffness of the results is plotted on Fig. 8, where we can notice the increased composite efficiency for the fibre coaxial to load series. The resulting behaviour of the specimen groups is plotted onto an Ashby map and presented in Fig. 9. The composites display an average performance as compared to the MBC state-of-the-art, and interestingly consolidate the existence of a material pole situating in between foam and elastomer behaviour, as per Young’s modulus. Commercial applications for such materials typically situate as EPS or XPS sustainable alternatives for insulation or packaging applications, while the elastic modulus of EPS is 6.5–265 MPa for a yield strength of 0.04–10.9 MPa. For its biochemical profile and impact on phylogenetic mycelial expression, or mechanical interest both at the scale of the particles or engineered artefact, heterogeneous and functionalised substrate design for MBC is a scarcely studied yet promising field of research towards market-ready sustainable and creative applications.
